# Role of the stability of charge ordering in exchange bias effect in doped manganites

**DOI:** 10.1038/s41598-017-03451-z

**Published:** 2017-06-12

**Authors:** Papri Dasgupta, Kalipada Das, Santanu Pakhira, Chandan Mazumdar, S. Mukherjee, S. Mukherjee, A. Poddar

**Affiliations:** 1 CMP Division, Saha Institute of Nuclear Physics, 1/AF, Bidhannagar, Kolkata 700 064 India; 2 Indian Association for the Cultivation of Science, 2A and 2B Raja S. C. Mullick Road, Jadavpur, Kolkata 700032 India; 3 UGC-DAE Consortium for Scientific Research, Mumbai Centre, Bhabha Atomic Research Centre Campus, Trombay, Mumbai 400 085 India; 4 Department of Physics, The University of Burdwan, Golapbag, Burdwan, West Bengal India; 5 Department of Physics, Seth Anandram Jaipuria College, 10 Raja Naba Krishna Street, Kolkata, 700005 India

## Abstract

In this work we have carried out an elaborate study on the magnetic properties and investigated the exchange bias phenomena of some charge-ordered (CO) manganites. The detailed study of Sm_1−*x*_Ca_*x*_MnO_3_ (x = 0.5, 0.55, 0.6, 0.65, 0.7) compounds shows that Sm_0.4_Ca_0.6_MnO_3_, which is the most robust charge ordered material studied here, shows significantly large exchange bias field (H_E_) as compared to the other compounds. Our experimental results and analysis indicate that T_CO_, which reflects the stability of the charge-ordered state, is one of the key parameters for the exchange bias effect. Similar behaviour is found in other rare-earth analogues, *viz*., La_1−*x*_Ca_*x*_MnO_3_ and Pr_1−*x*_Ca_*x*_MnO_3_ compounds as well. We also found that with increasing stability of CO states in Sm_1−*x*_Ca_*x*_MnO_3_ compounds, H_E_ enhances due to increase in number and reduction in size of ferromagnetic clusters.

## Introduction

Ferromagnets, under exposure to a cyclic magnetic field, exhibit a magnetic hysteresis loop (*M-H* loop) resulting in energy storage in the system^1^. Such loops appear due to irreversible domain wall motion/domain rotation caused by various pinning centres like dislocations and inclusions present in the system. In the permanent magnets, *M-H* loops are generally symmetric in nature with appreciable remnant magnetization and coercive field. Isothermal magnetic hysteresis phenomenon has been utilized in many technological applications, such as data processing, electronic, automobile, aerospace and biosurgical industries *etc*.^2^. However, for a few material, M-H loops have been found to be asymmetric in magnetic field direction, and such an asymmetry has been assigned to the presence of exchange bias (EB) phenomenon^3, 4^. The asymmetry parameter is called exchange bias field (H_E_) and is defined as (H_*C*1_ − H_*C*2_)/2 where H_*C*1_ and H_*C*2_ are coercive fields in the positive and negative field axis. EB is commonly observed in systems that contain phase boundaries separating antiferromagnetic (AFM) and ferromagnetic (FM) regions^3^. The exchange interaction at such magnetic phase boundaries would lead to unidirectional pinning of interfacial spins. In this case, reversal of the oriented FM moment needs an additional energy, leading to the shift in the switching field, thereby introducing asymmetry in M-H loop. Systems with large H_E_ have found several application in different magnetic sensors and read-heads for spintronic applications^5, 6^. EB is observed in various configurations viz, core-shell nanostructures, layered structures, two phase nanocomposits, inhomogenous materials *etc*.^4, 7–9^. In all these cases, artificially engineered phase boundaries between two or more crystallogaphically or chemically different phases have been used to achieve the magnetic inhomogenity required for introducing EB. However, such multiple-phase-compounds often found to exhibit compositional variations in the physical scale of micron or sub-micron range, which may affect the reproducibility of EB data measured in different batches of same material^10^. There are, nevertheless, some single phase compounds such as manganites, cobaltates, *etc*., known to exhibit EB, where magnetic inhomogenity are an inherent characteristics originating due to electronic phase separation^11^.

Electronic phase separation (hence onwards referred as phase separation in rest of this paper) has been observed and studied extensively in partially doped manganites^11–13^. Such doped manganites R_1−*x*_A_*x*_MnO_3_ (R = Rare-earth) are obtained by substituting divalent alkaline-earth ions such as A = Ca^2+^, Ba^2+^ and Sr^2+^ at the trivalent rare earth site of the parent RMnO_3_^13^. In the parent compound, the manganese ions exist in Mn^3+^ state whereas in doped compounds both Mn^3+^ and Mn^4+^ ions exist in the ratio of (1-x): x. This mixed-valent character of Mn ions underlies the rich phase diagrams exhibited with different ground states^13^. For example, one may get metallic or insulating state as well as FM, AFM, canted AFM or charge-ordered (*i.e*. a periodic arrangement of Mn^3+^ and Mn^4+^ ions in the lattice) states depending on the percentage of doping^13^. The presence of more than one of such electronic phases in an otherwise chemically homogenous system gives rise to phase separation. In phase separated manganites, EB was first reported in charge-ordered (CO) compound Pr_1/3_Ca_2/3_MnO_3_^14^. In this CO system, the regular arrangement of Mn^3+^ and Mn^4+^ ions also gives rise to AFM ordering. This CO/AFM system, sometime found to contain small inclusions of slightly different electronic phases, giving rise to FM behaviour in the form of nanodomains^14^. Since the exchange bias originate from the pinned interface spins between adjacent FM and AFM domains, H_E_ depends on the surface area of the FM clusters dispersed in CO/AFM matrix. Thus, it is expected that in a bulk compound, H_E_ should increase as the surface area of the FM clusters increases. This can be achieved by decreasing their size and increasing their numbers. To have large H_E_ in the bulk system, such large surface area is essential and can be obtained by arresting the growth of FM cluster size. This is possible in a robust CO system where the strong AFM interaction would restrict the size and growth of the FM clusters. The restriction imposed by the charge conservation would lead to an increase in the number of FM clusters of very small sizes throughout the material. Therefore, in this work, we propose that the robustness/stability of CO system, that is defined by the magnetic field required to melt the CO-state, must be an important parameter in deciding the value of H_E_ in bulk CO manganite. Earlier studies on R_1−*x*_Ca_*x*_MnO_3_ (R = Pr-Sm) compounds showed that the CO melting field and charge ordering temperature (T_CO_) increases with heavier rare earth analogues, indicating increase in the stability of CO state for a particular doping concentration^15, 16^. The largest value of CO melting field in the R_1−*x*_Ca_*x*_MnO_3_ has been reported so far in Sm_0.5_Ca_0.5_MnO_3_ (~60 T) suggesting it to be the strongest CO system studied till now^16^. Furthermore, for a particular rare earth, the CO melting field *i.e*. the strength of CO, increases with increasing T_CO_ (see Fig. 13 of ref. 14). So, one would expect even larger CO melting field in Sm_0.4_Ca_0.6_MnO_3_ (reportedly having maximum value of T_CO_ in Sm_1−*x*_Ca_*x*_MnO_3_ series^17^), though no such studies have been reported in literature. In this work, we focus on Sm_1−*x*_Ca_*x*_MnO_3_ (x = 0.5, 0.55, 0.6) and study the evolution of H_E_ with x. We show that the bulk Sm_0.4_Ca_0.6_MnO_3_ compound which has relatively larger CO stability, indeed exhibit considerably higher value of H_E_ than that observed in Sm_0.5_Ca_0.5_MnO_3_. Furthermore, H_E_ is found to reduce drastically in nanosized material of Sm_0.4_Ca_0.6_MnO_3_, where the CO strength is remarkably weakened. We have also studied H_E_ values of R_1−*x*_Ca_*x*_MnO_3_ (R = La, Pr) for compositions having maximum T_CO_ and compared these values with that of Sm_0.4_Ca_0.6_MnO_3_ compound to test the validity of our proposal.

## Results and Discussion

The bulk samples of Sm_1−*x*_Ca_*x*_MnO_3_ (x = 0.5, 0.55, 0.6, 0.65, 0.7) and nano-sized samples of Sm_0.4_Ca_0.6_MnO_3_ (Sm_0.4_Ca_0.6_MnO_3_-nano) have been prepared by sol-gel technique. All the peaks in the X-ray diffraction (XRD) patterns of these compounds at room temperature could be indexed by orthorhombic structure with *Pnma* space group, suggesting single phase nature of the samples (details are given in the section 1 of Supplementary information part). The lattice parameters obtained for x = 0.5, 0.55 and 0.6 compositions are *a* = 5.364 Å, 5.355 Å and 5.349 Å, *b* = 7.561 Å, 7.552 Å and 7.532 Å, *c* = 5.420 Å, 5.404 Å and 5.387 Å, respectively.

**Figure 1:**
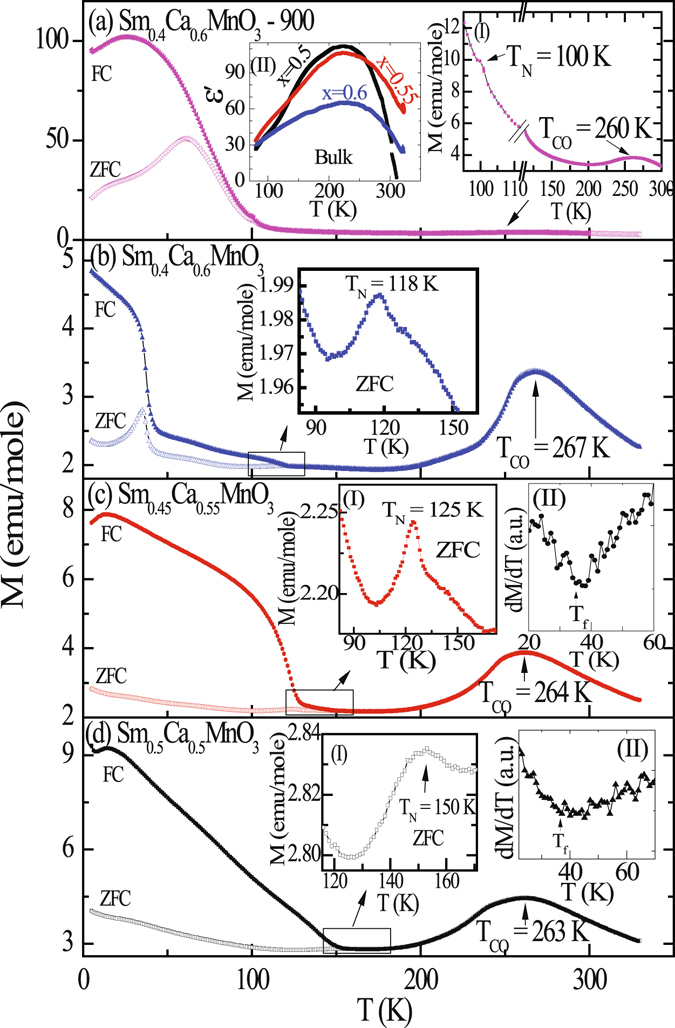
The temperature dependent magnetization of bulk Sm_1−*x*_Ca_*x*_MnO_3_ (x = 0.5, 0.55 and 0.6) and Sm_0.4_Ca_0.6_MnO_3_-nano compounds at H = 100 Oe. Inset I of (**a**), shows the enlarged view of the magnetization M(T) to point out the charge ordering signature and AFM ordering present even in nanoparticle. Inset II of (**a**) shows the dielectric constant of bulk Sm_1−*x*_Ca_*x*_MnO_3_ (x = 0.5, 0.55 and 0.6) compounds. Inset of (**b**) and insets I of (**a**,**c**,**d**) shows the enlarged view of the magnetization M(T) close to their respective AFM ordering temperatures. Insets II of (**c**,**d**) show the derivative curve of M(T).

Temperature dependence of magnetization, M(T), of all these samples studied in the temperature range of 5 K to 330 K under zero field cooled (ZFC) and field cooled (FC) conditions at H = 100 Oe are shown in Fig. 1(a–d). For all the samples, M(T) shows a monotonously increasing behavior as the temperature is lowered from 330 K to ~270 K till a peak is observed at T_CO_ ~ 270 K. It has been argued that although the material remains paramagnetic both above and below T_CO_, the magnetic interaction changes from FM (T > T_CO_) to AFM (T < T_CO_) at the charge ordering temperature^18^. This results in a peak-like structure at T_CO_ in the magnetic susceptibility. A close look into the M(T) data of Sm_0.4_Ca_0.6_MnO_3_-nano (Fig. 1a) reveals that the peak-like structure is very weak, indicating the suppression of CO as well as AFM interaction to a great extent. This results in a relative enhancement of magnetic susceptibility in the Sm_0.4_Ca_0.6_MnO_3_-nano at low temperatures vis-à-vis that of corresponding bulk materials. Charge ordering feature in these manganites is also manifested through the occurrence of an anomaly in dielectric constant around T_CO_ (Inset I: Fig. 1a). Generally, it has also been reported that in similar systems CO is followed by CE-type insulating AFM phase at lower temperature^16^. This is also observed in our systems where the M(T) curves exhibit a discernible hump indicating AFM ordering below T_CO_ (insets I of Figs. 1a,c,d and inset of Fig. 1b). Below the AFM ordering, M(T) under ZFC and FC configuration exhibit irreversible behavior (T_*irr*_ ~ T_*N*_). With the increasing magnetic field, the magnetic irreversibility reduces. Observation of such magnetic irreversibility in an AFM material suggests the presence of additional FM interaction. Below T_*N*_, ZFC M(T) curve for both the bulk and nano samples measured at a very low field of 100 Oe show an increasing trend with decreasing temperature indicating that the FM interactions are spontaneous and not field induced. The magnetization value of Sm_0.4_Ca_0.6_MnO_3_-nano below T_*N*_ appears to be quite high relative to the bulk compounds. Such an enhancement of magnetization in nano material is due to increasing ferromagnetic component arising from the uncompensated surface spins in comparison with the AFM core.

On further decreasing the temperature, Sm_0.4_Ca_0.6_MnO_3_ shows another cusp in the M(T) curve at 35 K (~T_*f*_), much below T_*N*_ (Fig. 1c). This low temperature cusp, observed earlier in quite a few manganite systems had been argued due to the cluster glass behaviour, where T_*f*_ represents the spin freezing temperature^19, 20^. Among all the compounds studied here, such spin freezing effect, although quite prominent for Sm_0.4_Ca_0.6_MnO_3_, found to be present in other bulk compounds as well. The temperature derivative of M(T) exhibit an anomaly around T_*f*_ indicating the spin freezing behaviour in the Sm_1−*x*_Ca_*x*_MnO_3_ (x = 0.5, 0.55) bulk compounds studied here (inset II: Fig. 1c,d). This effect, however, has not been observed in Sm_0.4_Ca_0.6_MnO_3_-nano, within the resolution limit of measurement.

Another important fact should mention here which is the effect of the external magnetic field on the transport properties of robust charge-ordered compound. In Sm_1−*x*_Ca_*x*_MnO_3_ (x ~ 0.5), since the charge ordering state is very robust, the insulating state at the low temperature is almost unaffected even in the presence of 90 kOe applied magnetic field. The resistivity as a function of temperature of some selected compounds in the absence and in the presence of magnetic field (90 kOe) is shown in supplementary part.

**Figure 2:**
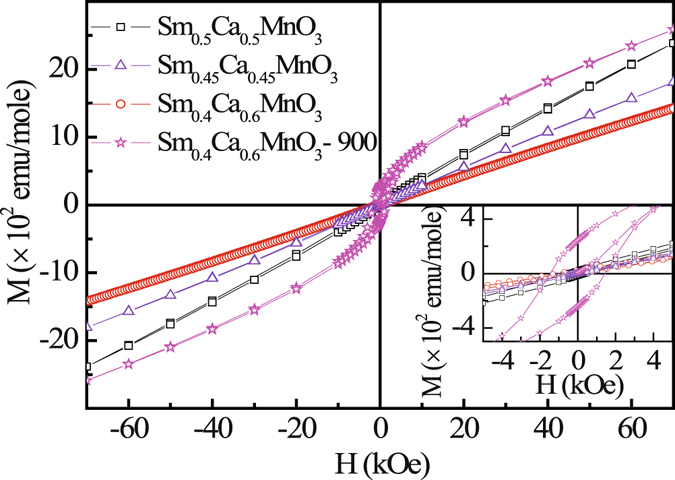
Magnetization as a function of external magnetic field of bulk Sm_1−*x*_Ca_*x*_MnO_3_ (x = 0.5, 0.55, 0.6) and Sm_0.4_Ca_0.6_MnO_3_ - nano compounds at T = 5 K. Inset shows the enlarged view of M(H) curves for low field region.

Field dependence of the magnetization, M(H), under ZFC condition for the bulk Sm_1−*x*_Ca_*x*_MnO_3_ (x = 0.5, 0.55, 0.6) and Sm_0.4_Ca_0.6_MnO_3_-nano samples at 5 K are shown in Fig. 2. The M(H) loops are symmetric and show an almost linear behavior in the high field region indicating a dominance of the AFM contribution in all the samples. A closer look at the low field region exhibit a weak loop in the M(H) curve suggesting the presence of an additional weak FM interaction (inset: Fig. 2). The remanence (M_*r*_) and coercivity (H_*C*_) parameters are of the order of ~25 emu/mole and ~550 Oe, respectively for the bulk samples. Sm_0.4_Ca_0.6_MnO_3_-nano shows much larger values of M_*r*_ (~243 emu/mole) and H_*C*_ (~1450 Oe) as compared to the corresponding bulk compound indicating relatively stronger FM interaction and weaker AFM interaction, as also inferred from M(T) studies. The relatively weak AFM interaction in Sm_0.4_Ca_0.6_MnO_3_-nano, makes the overall magnetization value high compared to its bulk counterpart. The loop could not be observed in the M(H) curve for the bulk samples at higher temperature (T > T_*N*_), *e.g*. at 150 K and 250 K, but instead show linear behavior with negligible M_*r*_ and H_*C*_. Thus, opening of the loop in the M(H) curve at 5 K indicates the presence of small FM clusters in AFM background, that has also been inferred from M(T) studies discussed earlier.

**Figure 3:**
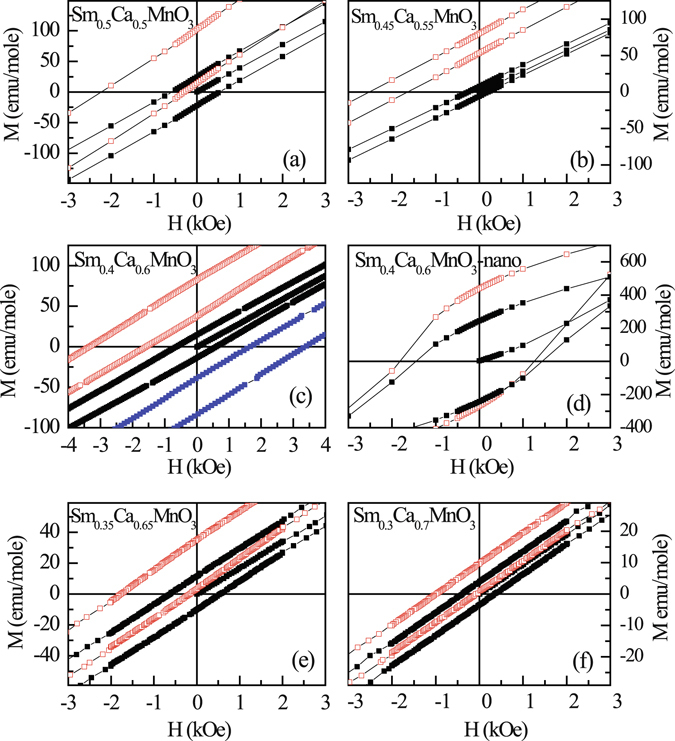
Magnetization as a function of external magnetic field for (**a**–**c**,**e**,**f**) bulk Sm_1−*x*_Ca_*x*_MnO_3_ (x = 0.5, 0.55, 0.6, 0.65 and 0.7) and (**d**) Sm_0.4_Ca_0.6_MnO_3_ - nano compounds at T = 5 K in ZFC (black symbol) and FC (red symbol) at H = 50 kOe, conditions used to measure exchange bias effect. An additional M(H) data for Sm_0.4_Ca_0.6_MnO_3_ compound at T = 5 K in FC (H = −50 kOe) protocol is also shown in (**c**) (denoted by blue symbol).

**Figure 4:**
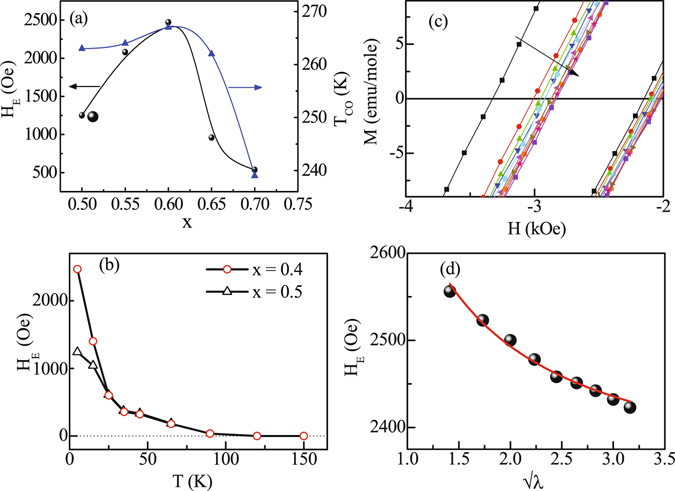
(**a**) Variation of H_E_ and T_CO_ as a function of Ca-concentration (x). (**b**) Variation of H_E_ with temperature in bulk Sm_1−*x*_Ca_*x*_MnO_3_ compounds for x = 0.5 and x = 0.6. (**c**) M(H) loops showing training effect of exchange bias and (**d**) Variation of H_E_ with the loop index number (*λ*) in Sm_0.4_Ca_0.6_MnO_3_ compound.

To check the presence of EB in these materials, M(H) measurements under FC condition *i.e*. after cooling the samples in a field of 50 kOe, have been carried out on bulk Sm_1−*x*_Ca_*x*_MnO_3_ (x = 0.5, 0.55, 0.6, 0.65, 0.7) and Sm_0.4_Ca_0.6_MnO_3_-nano samples at 5 K (Fig. 3a–f). M(H) loop of all the bulk samples (Fig. 3a–c, e, f) are found to be asymmetric about both the field as well as magnetization axis indicating the presence of EB effect. The asymmetry is so large that the values of M_R2_ are never positive even for large reverse fields of −70 kOe, where M_R1_ and M_R2_ are remanence magnetization corresponding to the decreasing and increasing field cycling of M-H loop. This indicates the presence of strong exchange anisotropy in all these compounds. To further confirm the EB effect in these compounds, additional measurements were carried out on bulk Sm_0.4_Ca_0.6_MnO_3_ that exhibits largest H_E_ value among all the compounds studied in this work. M(H) loop, measured at 5 K after cooling the samples in a field of -50 kOe, shifted to the positive field direction indicating that the shift in *M-H* is not an artifact but is occurring due to EB effect (Fig. 3c). An important property usually observed in the EB system is the magnetic training effect in which the H_E_ and remanence asymmetry (M_E_ = (M_R1_ + M_R2_)/2) values are known to decreases as the system undergoes successive field cycling at a particular temperature after field cooling^19^. Furthermore, the variation of H_E_ with the loop index number (*λ*) follows the relation (H_E_ − H_E∞_) ∝ 1/√*λ*, where H_E∞_ is the EB field at *λ* = ∞^19^. It has been observed that similar behaviour is followed in bulk Sm_0.4_Ca_0.6_MnO_3_ compound when field cycled at 5 K (Fig. 4c,d), thus confirming the presence of exchange bias effect in bulk Sm_1−*x*_Ca_*x*_MnO_3_ compounds studied here. The estimated H_E_ values found to be ~1250 Oe, ~2080 Oe, ~2470 Oe, ~960 Oe and ~537 Oe at 5 K for these bulk samples with x = 0.5, 0.55, 0.6, 0.65 and 0.7 respectively. Thus, H_E_ values of the bulk Sm_1−*x*_Ca_*x*_MnO_3_ (x = 0.5, 0.55, 0.6, 0.65, 0.7) compounds qualitatively mimics the variation of T_CO_ with x similar to the reported phase diagram^17^ (Fig. 4a). Furthermore, these compounds show large H_E_ values. On the contrary, Sm_0.4_Ca_0.6_MnO_3_ - nano does not show any loop shift (H_E_ ~ 0 Oe), indicating that H_E_ vanishes as CO is suppressed drastically (Fig. 3d). Furthermore, it is known from previous studies that CO stability and T_CO_ increases from R = La to Sm in R_0.5_Ca_0.5_MnO_3_ series^15^, and for a particular R-ion CO stability increases with increasing T_CO_ (see Fig. 13 of ref. 14). Accordingly we have estimated the H_E_ values of La_0.32_Ca_0.68_MnO_3_ and Pr_0.4_Ca_0.6_MnO_3_ compounds that have the largest T_CO_ (most stable CO for a particular rare earth) in the La_1−*x*_Ca_*x*_MnO_3_ and Pr_1−*x*_Ca_*x*_MnO_3_ series respectively^11^. The H_E_ values of these two compounds found to be 625 Oe and 1200 Oe which are much less than that of Sm_0.4_Ca_0.6_MnO_3_ (H_E_ ~ 2470 Oe) supplementing our proposal that H_E_ increases with increasing CO stability.

To reveal the origin of EB effect and the increase in the values of H_E_ with increasing CO stability and x value in Sm_1−*x*_Ca_*x*_MnO_3_ (x = 0.5, 0.55, 0.6) system, the temperature dependence of H_E_ has been studied (Fig. 4b). H_E_ obtained for Sm_0.4_Ca_0.6_MnO_3_ compound up to a temperature of 150 K shows that above T_*irr*_, H_E_ becomes temperature independent with a negligible value. Below T_*irr*_, H_E_ increases slowly with decreasing temperature and then rises at a much faster rate below the spin freezing temperature T_*f*_ ~ 35 K. Similar variation of H_E_ is also observed for Sm_0.5_Ca_0.5_MnO_3_ compound (Fig. 4b) which too exhibits a signature of spin freezing in the M(T) curve as shown in inset II of Fig. 1d. Increase in H_E_ below 35 K was also observed previously in Pr_1/3_Ca_2/3_MnO_3_ manganite system (which do not exhibit any prominent cusp at low temperature) and was ascribed to the glassy behavior of the compound^14^. This indicates that the EB effect originates below T_*irr*_ (~T_*N*_), *i.e*. with the formation of FM clusters and increases drastically below freezing temperature in all the bulk Sm_1−*x*_Ca_*x*_MnO_3_ (x = 0.5, 0.55, 0.6) compounds. To confirm the presence of ferromagnetic clusters and glassy behavior further measurements, as described below, have also been carried out.

**Figure 5:**
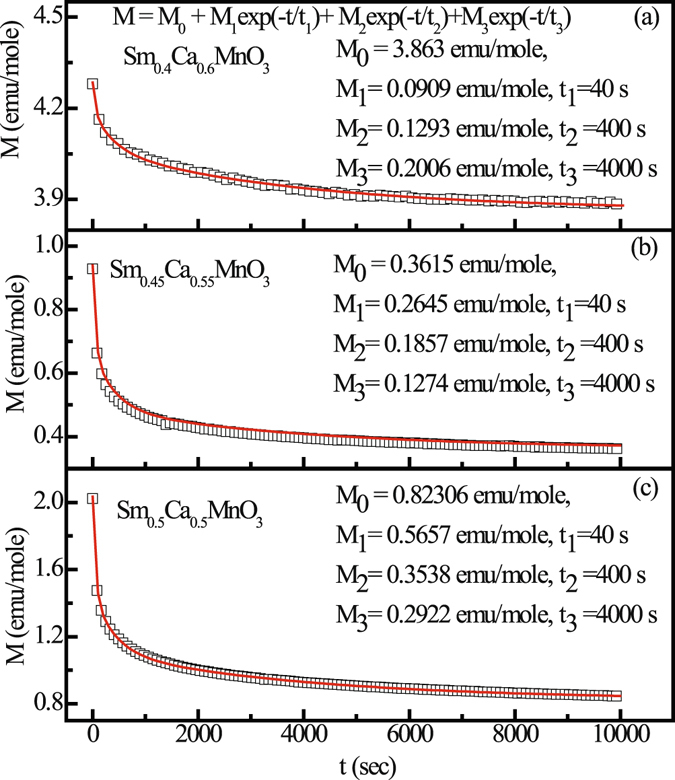
M as a function of time t for bulk Sm_1−*x*_Ca_*x*_MnO_3_ (x = 0.5, 0.55 and 0.6) compounds at 5 K. Solid line is a fit to M = M_0_ + M_1_exp(−t/t_1_) + M_2_exp(−t/t_2_) + M_3_exp(−t/t_3_).

Magnetic relaxation experiments on Sm_1−*x*_Ca_*x*_MnO_3_ (x = 0.5, 0.55, 0.6) were carried out to confirm the presence of FM clusters. We have measured the isothermal remnant magnetization (IRM) as a function of time at T = 5 K, after cooling the sample in ZFC condition, followed by cycling in an external field of 0 Oe → 1 kOe → 0 Oe. M(t) data for Sm_1−*x*_Ca_*x*_MnO_3_ (x = 0.5, 0.55, 0.6) shown in Fig. 5(a–c), relaxes as M(t) = M_0_ + M_1_ exp(−t/t_1_) + M_2_exp(−t/t_2_) + M_3_exp(−t/t_3_), where M_0_ is the time independent and M_1_, M_2_, M_3_ are three time dependent fractions relaxing with time constants t_1_, t_2_, and t_3_, respectively^21^. It can therefore be said that our relaxation experiment confirms the presence of multiple relaxation times, as expected for a collection of FM clusters.

**Figure 6:**
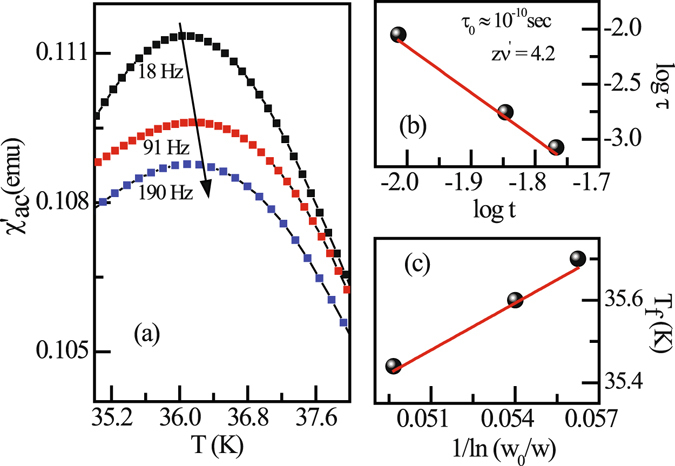
(**a**) The temperature dependence of the real parts of the ac magnetic susceptibility of Sm_0.4_Ca_0.6_MnO_3_ compound measured at different frequencies in an applied ac magnetic field of 3 Oe. (**b**) The frequency dependence of freezing temperature plotted as a log(*τ*) *vs*. log(t). (**c**) The frequency dependence of freezing temperature plotted as T_*f*_ *vs*. 1/ln(*ω*_0_/*ω*).

The presence of FM clusters in an antiferromagnetic matrix is expected to exhibit glassy behavior. To confirm the glassy nature of the system, the ac susceptibility measurement has been carried out for the compound Sm_0.4_Ca_0.6_MnO_3_ at different frequencies. The ac susceptibility exhibit a peak around 35 K, and with increase in frequency the peak temperature shifts towards higher temperatures (Fig. 6(a)). This type of frequency dependence of magnetic transition is typical for glassy systems and the corresponding peak temperature as *f* → 0 is defined as the freezing temperature. In typical glassy system, the relative shift in freezing temperature per decade of frequency is determined by ref. 22

1 $$\delta {T}_{f}={\rm{\Delta }}{T}_{f}/{T}_{f}{\rm{\Delta }}({\mathrm{log}}_{10}\nu )$$

The calculated value of *δ*T_*f*_ is found to be ~0.12 for the compound, which is typically in the range of that found in cluster glass systems^23^. The frequency dependence of the glassy systems also follows conventional power-law divergence of critical slowing down

2 $$\tau ={\tau }_{0}{[({T}_{f}-{T}_{SG})/{T}_{SG}]}^{-z\nu ^{\prime} }$$

where *τ* is relaxation time corresponding to the measured frequency (*τ* = 1/*ν*), *τ*_0_ is single spin flip relaxation time, T_*SG*_ is the freezing temperature corresponding to zero frequency and *zν*′ is the dynamic critical exponent. The estimated value of *zν*′ is 4.2 and *τ*_0_ ≈ 10^−10^ sec [Fig. 6(b)] from the ac susceptibility data again found to be in the same range to those of cluster glass compounds^24^.

Another dynamical law for glassy systems is the Vogel-Fulcher law,

3 $$\nu ={\nu }_{0}\,\exp (-\frac{{E}_{a}}{{K}_{B}({T}_{f}-{T}_{0})})$$

where *ν*_0_ is the attempt frequency, E_*a*_ is the activation energy and T_0_ is the Vogel-Fulcher temperature. The best fitted experimental data yields E_*a*_/K_*B*_ = 38.9 K and T_0_ = 33.6 K [Fig. 6(c)].

The glassy feature may also be demonstrated through dc magnetization measurement *viz*. memory effect as well as magnetic relaxation. Memory effect has been observed in bulk Sm_0.4_Ca_0.6_MnO_3_ compound [see Supplementary for full information]. The presence of cluster glass state in this compound is further proved by the analysis of temperature dependent magnetic relaxation data. According to Ulrich *et al*.^25^, the relaxation rate of remanent magnetization $[{\rm{W}}({\rm{t}})=-\frac{d}{dt}\,{\rm{l}}{\rm{n}}\,{\rm{M}}({\rm{t}})]$ in a system consisting of magnetic clusters obey the decay law, W(t) = Bt^−*n*^, t ≥ t_0_, where B is a constant, t_0_ is crossover time and ‘*n*’ is a measure of the dipolar interaction strength among the magnetic clusters involved in the relaxation process.

**Figure 7:**
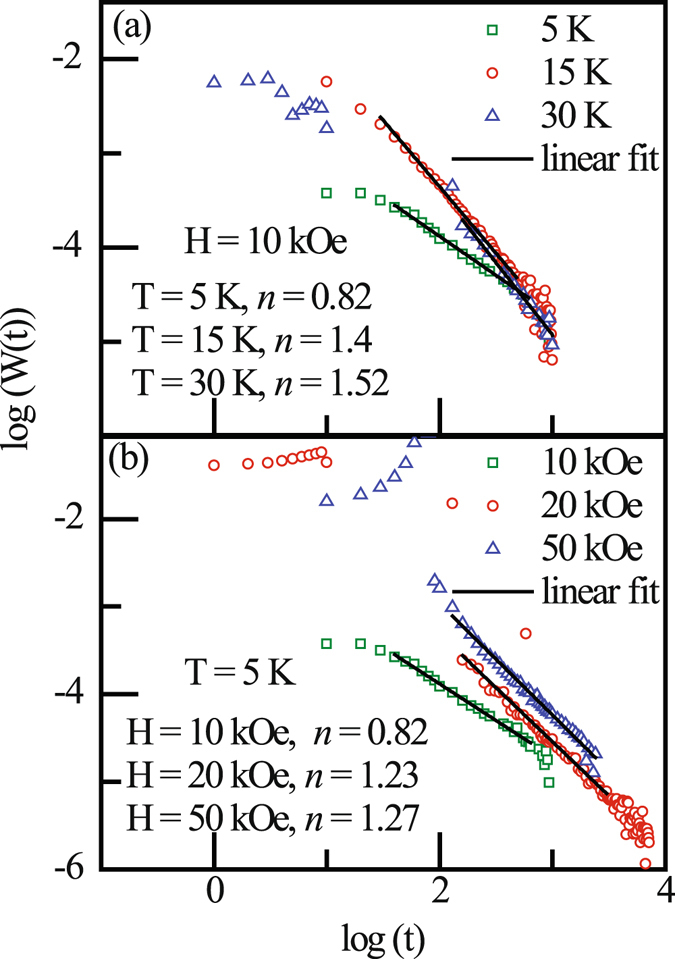
Relaxation rate W(t) for bulk Sm_0.4_Ca_0.6_MnO_3_ compound with (**a**) variation of temperature and (**b**) variation of field.

In case of canonical spin glass, the value of ‘*n*’ remain almost constant, whereas it changes in case of cluster glass systems. In our case, ‘*n*’ is found to be sensitive to both temperature as well as applied magnetic field (Fig. 7a,b). Such dynamic behaviour ‘*n*’ is also found for other two bulk compounds studied here. The fraction of glassy component in this compound thus certainly not static but found to be dependent on both temperature and magnetic field, thus confirming the presence of cluster glass state in bulk Sm_1−*x*_Ca_*x*_MnO_3_ compounds.

All these results firmly establish cluster glass behaviour with the freezing of FM spins below T_*f*_ in the bulk Sm_1−*x*_Ca_*x*_MnO_3_ (x = 0.5, 0.55, 0.6) compounds. The interface exchange coupling between these FM clusters and the CO AFM background lead to unidirectional shift of the hysteresis loops *i.e*. EB effect. Additionally, we have also seen that H_E_ depends strongly on x where H_E(*x*=0.4)_ ~ 2 H_E(*x*=0.5)_ at 5 K. To understand the increase of H_E_ with x, we have estimated the relative FM and AFM/PM fraction from the isothermal magnetization data using the relation, M(H) = *P* tanh(*Q*H) + *S*H, where, *P*, *Q* and *S* are constants. The fraction remains almost conserved in all the three compounds suggesting that the FM volume fraction is not responsible for the increase of H_E_ with increasing x. The above result leads to investigate the number and size distribution of the clusters with variation of x. Since H_E_ is known to depend primarily on the FM and AFM interface area rather than the FM volume fraction, a system consisting of smaller clusters in larger number is expected to exhibit larger H_E_ than systems with larger clusters with smaller number.

**Figure 8:**
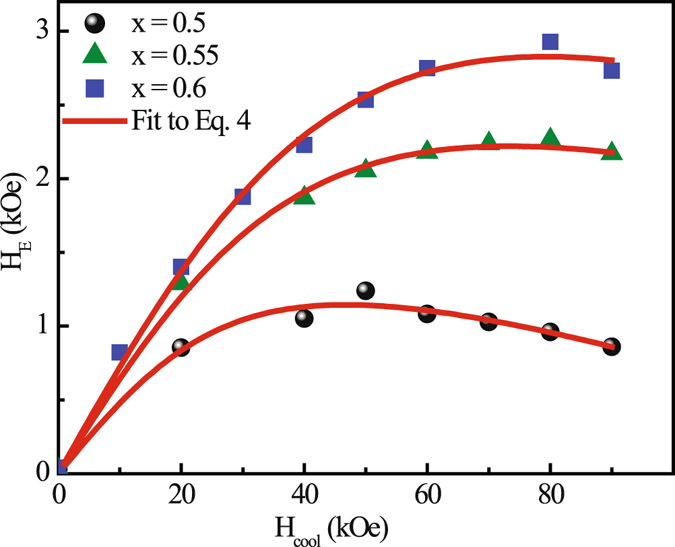
Variation of H_E_ with cooling magnetic field. Red lines represent the fitting using relation 4 for Sm_1−*x*_Ca_*x*_MnO_3_ (x = 0.5, 0.55 and 0.6) bulk compounds.

**Figure 9:**
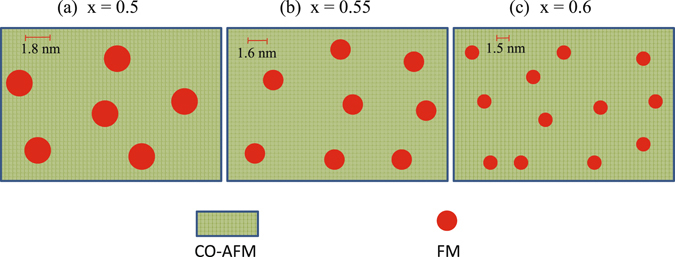
Schematic diagram showing variation of size and density of ferromagnetic clusters in phase co-existing charge ordered Sm_1−*x*_Ca_*x*_MnO_3_ (x = 0.5, 0.55 and 0.6) compounds.

The average size of short range ferromagnetic clusters can be generally estimated following the formula given by Niebieskikwiat and Salamon in several phase separated manganite and cobaltite systems^14^. According to them, the cooling field (H_cool_) dependence of H_E_ can be expressed by the relation,

4 $$-{H}_{E}\propto {J}_{i}[\frac{{J}_{i}{\mu }_{0}}{{(g{\mu }_{B})}^{2}}L(\frac{\mu {H}_{cool}}{{K}_{B}{T}_{f}})+{H}_{cool}]$$

where *J*_*i*_ is the interface exchange constant, *g* ≈ 2 is the gyromagnetic factor, *μ*_*B*_ is the Bohr Magneton, *μ*_0_ ≈ 3*μ*_*B*_ is the magnetic moment of the Mn core spin, *L* is the Langevin function, *μ* = N_*ν*_ *μ*_0_ is the magnetic moment of ferromagnetic cluster, N_*ν*_ is the number of spins in a cluster and T_*f*_ is the temperature below which glassy state exists. The first term in relation 4 dominates for small H_cool_ and H_E_ depends on *J*_*i*_^2^. However the second term dominates for large H_cool_ and for J_*i*_ < 0 the value of H_E_ decreases for higher H_cool_. M(H) measured at different cooling fields (H_cool_) for bulk Sm_1−*x*_Ca_*x*_MnO_3_ (x = 0.5, 0.55, 0.6) samples (Fig. 8) shows that H_E_ initially increases with H_cool_ and thereafter exhibits a decreasing tendency above H_cool_ ~ 8 T. Figure 8 show the H_E_ as a function of H_cool_ along with the fit to Eq. 4 with an overall scale factor and J_*i*_, N_*ν*_ as adjustable parameters for all the bulk compounds. The exchange constant obtained from the best fit to the experimental data yields to be negative for all the samples, indicating the existence of FM clusters in AFM host (similar to that discussed earlier). For Sm_0.5_Ca_0.5_MnO_3_ compound, we have deduced the value of N_*ν*_ ≈ 14. We have also estimated the value of N_*ν*_ from the earlier reported H_*EB*_ *vs*. H_cool_ data for this particular system^19^ and found both the values of N_*ν*_ to be very similar. The value of N_*ν*_ systematically reduces to 10 for x = 0.55 and 8 for x = 0.6 compound. The average diameter of the FM clusters thus obtained are 18 Å, 16 Å and 14.9 Å derived using the lattice parameters of Sm_1−*x*_Ca_*x*_MnO_3_ (x = 0.5, 0.55, 0.6) compounds respectively. The density (N) of FM droplets for all the compounds is estimated from the relation M_*S*_ = N*μ*, where M_*S*_ is the saturation moment. The estimated values of ‘N’ are 3.6 × 10^−4^ Å^−3^, 5.4 × 10^−4^ Å^−3^ and 6.5 × 10^−4^ Å^−3^ for x = 0.5, 0.55 and 0.6 analogues, respectively. This analysis clearly shows that as the x value increases, the size of the FM clusters decreases while its density increases, which has been shown schematically in Fig. 9(a–c). For more generalization we have plotted the H_E_ as a function of Ca-doping even for higher Ca concentration than x = 0.6. Our experimental study clearly reveals that with the increasing of the Ca-doping up to x = 0.6 H_E_ increases, then decreases for x > 0.6 compositions qualitatively similar as T_CO_(x). Furthermore, using the size of FM clusters, exchange anisotropy constant can be calculated using the expression^14^

5 $$\frac{{M}_{E}}{{M}_{S}}\sim -2{\nu }_{0}\tau {e}^{(-KV/{K}_{B}T)}\,\sinh (\frac{\mu {H}_{E}}{{K}_{B}T})$$

where *ν*_0_ ~ 10^9^ s^−1^ is the switching attempt frequency, *τ* ~ 10^2^–10^3^ s is the typical measurement time, k_B_ is the Boltzmann constant, ‘V’ is the volume of FM clusters, *μ* is the magnetic moment of ferromagnetic clusters and ‘K’ is the anisotropy constant, M_S_ is the saturation magnetization. M_E_ = (M_R1_ + M_R2_)/2 values of Sm_1−*x*_Ca_*x*_MnO_3_ (x = 0.5, 0.55, 0.6) compounds are 58 emu/mole, 66 emu/mole, 60 emu/mole, respectively, estimated from FC *M-H* isotherm shown in Fig. 3. In the compounds studied here, the value of the anisotropy constant estimated at a temperature 5 K are 2.3 × 10^6^ erg/cm^3^, 3.3 × 10^6^ erg/cm^3^ and 4.1 × 10^6^ erg/cm^3^ for x = 0.5, 0.55 and 0.6, respectively. These values are considerably larger than that reported for bulk FM manganites^26^.

The effect of the variation of the size of FM clusters, i.e., the effect of magnetic anisotropy can be qualitative obtained using Meiklejohn and Bean model^7^, estimated for FM/AFM thin films using the relation originally proposed for PM/AFM multilayer thin films. According to this model, H_E_ can be expressed as

6 $${H}_{E}=-\frac{J{S}_{AFM}{S}_{FM}}{{\mu }_{0}{t}_{FM}{M}_{FM}}$$

where J is the exchange integral across the FM/AFM interface per unit area, S_*AFM*_ and S_*FM*_ are the interface magnetizations of the AFM and FM layers, respectively, and t_*FM*_ and M_*FM*_ are, respectively, the thickness and magnetization of the FM layer. By replacing the FM layer thickness with FM cluster size, the same model has been extensively used in phase separated manganite systems^27^. In our case, where FM clusters are dispersed in AFM matrix, as the size of FM clusters decreases, the term t_*FM*_ decreases. Furthermore, the decrease of cluster size with increasing x leads to the decrease in magnetization of the FM clusters. As both the terms in the denominator of Eq. (5) decreases with increasing x value, H_E_ increases. Hence, it can be shown qualitatively that with the decreasing size of FM clusters with corresponding increase in values of x in Sm_1−*x*_Ca_*x*_MnO_3_ (x = 0.5, 0.55, 0.6) compounds, H_E_ increases.

To conclude, we have correlated exchange bias field (H_E_) in CO manganites with the stability of charge ordering. For this, we have undertaken a detailed study of magnetic properties of CO Sm_1−*x*_Ca_*x*_MnO_3_ (x = 0.5, 0.55 and 0.6) bulk compounds and found that with increasing x value the T_CO_ (and hence stability of CO) and the corresponding H_E_ increases. Due to suppression of CO in nano form of Sm_0.4_Ca_0.6_MnO_3_, H_E_ is reduced drastically as compared to that in bulk. We have estimated the size and density of the FM clusters (which introduces EB in the CO AFM system) and found that with increasing x, exchange anisotropy constant and density of clusters increases while their size decreases. We have also found that H_E_ mimics the strength of CO stability in R_1−*x*_Ca_*x*_MnO_3_ as R changes from La to Sm. From our observation it appears that the increase in H_E_ with increasing CO stability may be a general behaviour for all CO systems and can be utilized in engineering large exchange bias materials.

## Methods

Polycrystalline samples of Sm_1−*x*_Ca_*x*_MnO_3_ (x = 0.5, 0.55, 0.6) have been prepared by well known sol-gel technique. Sm_2_O_3_, CaCO_3_ and MnO_2_ were used as starting materials. Appropriate amount of oxides were separately dissolved in a HNO_3_ solution (Oxalic acid is also added in case of MnO_2_). These solutions were mixed, and an amount of citric acid equivalent to the total number of moles of metal ions was added under moderate heating and stirring conditions. Subsequently mixture was slowly evaporated at 80–90 °C in water bath resulting in the formation of gel which was heated to 250 °C to remove the organic matter and decompose the nitrates of the gel. The black ash thus obtained was grinded and further heated to 550 °C for 5 h to kick off the remaining organic matter and then pelletized. The pellets then finally sintered for 24 hours at 1250 °C to obtain bulk samples. Nanoparticles of Sm_0.4_Ca_0.6_MnO_3_ (Sm_0.4_Ca_0.6_MnO_3_-900) has been prepared by sintering the pellets at 900 °C for 3 hours. X-ray diffraction (XRD) study was carried out, with Rigaku diffractometer in Brag-Brentano geometry, using Cu-K*α* source having wavelength 1.54 *Å*. Magnetization was measured using VSM-SQUID magnetometer (Quantum Design). Some of the Magnetization measurements were carried out on a vibrating sample magnetometer (VSM) coupled to a 9 Tesla physical property measurement system (PPMS) (Quantum Design, USA) at UGC-DAE Consortium for Scientific Research, Mumbai Centre.

## Electronic supplementary material

Supplementary Dataset 1
