# Supplementary material for: Role of the stability of charge ordering in exchange bias effect in doped manganites
Source: Sci Rep. 2017 Jun 12;7:3220. doi: 10.1038/s41598-017-03451-z (PMC5468231; doi:10.1038/s41598-017-03451-z)
Supplement: Supplementary file 1 — Supplementary Dataset 1 [file 41598_2017_3451_MOESM1_ESM.pdf]

Supplementary information part

Role of the stability of charge ordering in exchange bias effect in doped manganites

Papri Dasgupta[1*], Kalipada Das[2], Santanu Pakhira[1], Chandan Mazumdar[1], S. Mukherjee[3], S. Mukherjee[4], and A. Poddar[1]
[1] CMP Division, Saha Institute of Nuclear Physics, 1/AF, Bidhannagar, Kolkata 700 064, India
[2] Indian Association for the Cultivation of Science, 2A and 2B Raja S. C. Mullick Road, Jadavpur, Kolkata 700032, India
[3] UGC-DAE Consortium for Scientific Research, Mumbai Centre, BARC Campus, Trombay, Mumbai 400 085, India
[4] Department of Physics, The University of Burdwan, Golapbag, Burdwan, West Bengal, India

March 17, 2017

1 Supplementary information

1.1 X-ray diffraction

X-ray diffraction pattern of $Sm_{1-x}Ca_xMnO_3$ (x = 0.5, 0.55, 0.6, 0.65 and 0.7) bulk and $Sm_{0.4}Ca_{0.6}MnO_3$-nano samples have been carried out. All lines could be indexed with that permitted by the orthorhombic *Pnma* space group. The observed and calculated XRD patterns, allowed Bragg positions and the difference profiles from Rietveld refinement for all the compounds are displayed in Figure 1.

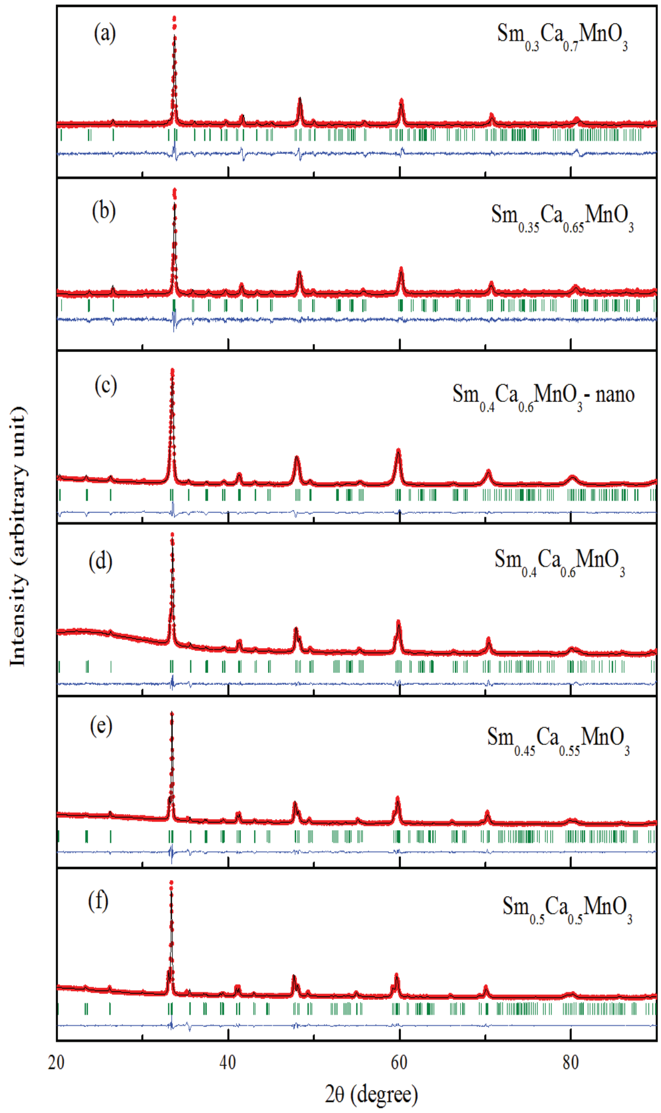

Figure 1: Room temperature x-ray diffraction pattern of the $Sm_{1-x}Ca_xMnO_3$ compounds having different x values ($x = 0.5$, 0.55, 0.6, 0.65 and 0.7) and $Sm_{0.4}Ca_{0.6}MnO_3$-nano samples. Red points are experimental and black line represents calculated patterns respectively. Green lines are allowed Bragg position by the space group. The difference of calculated and experimental data is shown in blue line.

1.2 Resistivity

Temperature dependence resistivity of some selected $Sm_{1-x}Ca_xMnO_3$ (x = 0.5, 0.6) compounds have been measured under zero field and 90 kOe applied magnetic field. No significant changes have been observed in the resistivity curves.

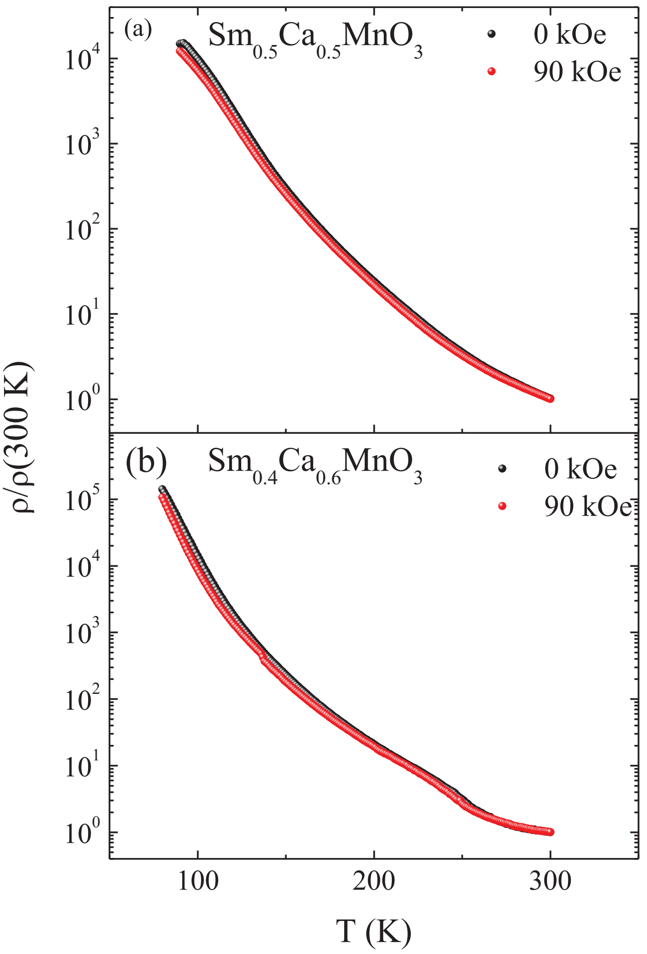

Figure 2: Temperature dependence of resistivity measured under zero field and at an applied magnetic field of 90 kOe.

1.3 Memory effect

Different kind of glassy systems is reported to exhibit a magnetic memory effect [1]. Magnetic memory effects in glassy magnetic systems can be measured using different protocols. In the $Sm_{0.4}Ca_{0.6}MnO_3$ system, the magnetic memory effect, if any, has been studied in the time variation of magnetization under the influence of temperature and field cycling on the relaxation behaviour in ZFC method using Sun *et al.* protocol [1]. In ZFC process, the sample is cooled down to a temperature $T_0(= 10$ K $< T_f)$ under zero field. After temperature stabilization at T_0, a small amount of magnetic field (100 Oe) is applied and time evolution of magnetization [M(t)] is measured for $t_1 = 1$ h, as shown in Fig. 2(a). After that, the sample is quenched to a temperature T_0-ΔT (5 K) in the absence of any magnetic field and M(t) is measured for $t_2 = 1$ h. Finally, the temperature is turned back to T_0 and after switching on the same magnetic field (100 Oe) and M(t) is measured for $t_3 = 1$ h. From fig.2(a) it is clear that the relaxation process during the time interval t_3 is nothing but the continuation to that of during t_1. This type of behavior concludes the presence of strong memory effect in the system and quite similar to that observed for several earlier reported glassy systems also [2, 3]. Fig. 2(b) shows the study of the memory effect on the magnetic relaxation behaviour similar process described above with intermediate heating ($T_0 + \Delta T = 15$ K) in the time interval $t_1 < t < t_2$. In this case the relaxation process during the time interval t_3 is not the continuation that of during t_1, thus temporary heating rejuvenates the relaxation process. This type of asymmetric response in the relaxation process favours the hierarchical model of memory effect rather than droplet model [1].

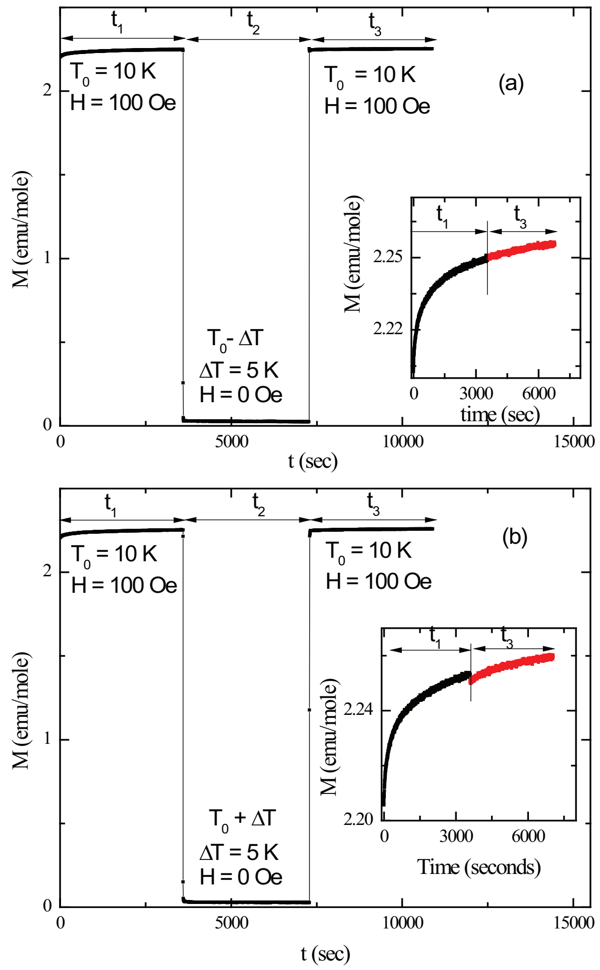

Figure 3: ZFC magnetic relaxation measured at 15 K and 100 Oe field with an intermittent decrease in temperature to 10 K with 0 Oe field. (b) ZFC magnetic relaxation measured at 10 K and 100 Oe field with an intermittent decrease in temperature to 5 K with 0 Oe field.

References

[1] Sun, Y., Salamon, M. B., Garnier, K. & Averback, R. S. Memory Effects in an Interacting Magnetic Nanoparticle System. Phys. Rev. Lett. 91, 167206(1)- 167206 (4) (2003).

[2] Pramanik, A. K. & Banerjee, A. The dynamics of magnetization in phase

separated manganite around half doping: A case study for Pr0.5Sr0.5Mn0.925Ga0.075O3. Phys. Lett. A 376, 996-1001 (2012).
[3] Giri, S. K., Yusuf, S. M., Mukadam, M. D. & Nath, T. K. Enhanced exchange bias effect in size modulated Sm0.5Ca0.5MnO3 phase separated manganite, J. Appl. Phys. 115, 093906(1)- 093906(10) (2014).
